# Physician perceptions and understanding of pet ownership in healthcare compliance and patient well-being: a one health investigation

**DOI:** 10.3389/frhs.2025.1620640

**Published:** 2025-07-23

**Authors:** Faith Kipnis, Emily McCobb, Megan K. Mueller, Meera Gatlin, Catharina A. Armstrong

**Affiliations:** ^1^ Cummings School of Veterinary Medicine, Tufts University, North Grafton, MA, United States; ^2^ Department of Medicine and Epidemiology, School of Veterinary Medicine, The University of California, Davis, CA, United States; ^3^ Department of Clinical Sciences, Cummings School of Veterinary Medicine, Tufts University, North Grafton, MA, United States; ^4^ Department of Infectious Disease and Global Health, Cummings School of Veterinary Medicine, Tufts University, North Grafton, MA, United States; ^5^ Department of Medicine, Newton-Wellesley Hospital, Newton, MA, United States

**Keywords:** human-animal bond, pet ownership, patient-centered care, healthcare compliance, communication, healthcare decision making

## Abstract

**Introduction:**

Pets contribute positively to human mental and physical health outcomes but can also impose financial obligations and personal responsibilities that may impede pet owners from accessing healthcare services, especially by restricting access to inpatient and long-term care. This preliminary study investigates the complex interplay between pet ownership and healthcare access and compliance through the perspective of physicians, exploring how pets influence their patients’ health.

**Methods:**

An online survey was designed and distributed to physicians in Massachusetts, USA. The survey collected information about participant demographics and their experiences and beliefs surrounding how pets influence physician-client relationships, healthcare access and compliance, and human-animal interactions.

**Results:**

Of 16 physicians surveyed from various specialties, 25% noted that they believe pets can be a barrier to accessing treatment or services among their patients. Many of the participants (56%) reported that they had experienced a patient who declined or resisted recommended treatments or services due to concerns about their pet. The most commonly reported barrier to healthcare was being unable to find pet care. Most participants (63%) agreed that a low or no-cost boarding facility could be an effective solution to address pet-related concerns among their patients. All participants reported that they believe that owning pets has psychosocial benefits.

**Discussion:**

Findings from this study indicate that there is a gap in resources available to patients with pet-related concerns that may prevent them from accessing healthcare services and that there is a need for more research on the role of pets in healthcare access and compliance.

## 1 Introduction

Pet ownership in the United States is widespread, with approximately 60% of the population owning at least one companion animal (1). Many pet owners consider their pets to be as important as any other family member (2), factoring into critical family decision-making and lifestyle choices. While pet ownership can contribute positively to mental health outcomes through companionship and unconditional support (3–7), there are also potential negative impacts associated with pet ownership that may impact human health outcomes. The most common identified risks are animal-related injuries (i.e., bite or scratch), exposure to zoonotic diseases, and certain social pressures, such as reduced housing access, increased emotional stressors, significant time commitment for care, and the financial burden of pet ownership (5, 6, 8). All of these factors may contribute to pet owners making sacrifices that can impact their own health and well-being. Previous research has found that pet owners may be more likely to avoid or delay accessing healthcare services, especially inpatient and long-term care, due to concerns about their pets (9–12). Strong attachment to a pet may result in a reluctance to prioritize one's own health interests if it conflicts with the human-animal bond. These concerns disproportionately impact individuals from vulnerable populations with limited social support and resources, such as low-income individuals, sexual and gender minorities, and unhoused individuals, as fear of being separated from one's pet may make them less willing to seek out medical care (12–15). Previous studies of unhoused pet owners suggest they are less likely to use medical care facilities and social services and are often forced to tighten their personal budgets to accommodate their pet's needs (14, 15). There is also evidence that pet owners may neglect their own safety by prioritizing the well-being of their pet; studies have found that victims of intimate partner violence may hesitate to leave their dangerous home environment due to concerns about their pet's safety (16). However, to our knowledge, there are no other studies that have examined how physicians perceive and manage pet ownership as a contributor to their patient's health outcomes, particularly when considering healthcare compliance and access.

This exploratory study aimed to assess physician perspectives on both the benefits and challenges associated with pet ownership in human healthcare settings, including how pets inform physician-client relationships, healthcare access and compliance, and human-animal relationships. Documenting the role of pets in healthcare decision-making and patient well-being could help enhance the clinical management of complex medical conditions in vulnerable populations. Specifically, it could establish the need for programs that support a patient's ability to receive appropriate treatment by addressing pet-related concerns. Findings from this preliminary study offer insights that can inform the development of projects and interventions to enhance patient care while accommodating pet care responsibilities.

## 2 Methods

The research team for this study included professionals from both veterinary and human medical backgrounds, providing multidisciplinary perspectives to the research.

### 2.1 Online survey of physicians

An online Qualtrics survey was disseminated to physicians in Massachusetts, USA from June to September 2024 (see Supplementary Material). Inclusion criteria included being a practicing physician in the Massachusetts area, including residents and research fellows. Physicians were recruited through convenience sampling via email invitation outlining the study's goals and the requirements for participating. The survey was developed by the research team with expert review from a physician, veterinarian, and PhD psychologist and was informed by previous literature (9, 11, 17). Since this was an exploratory study, survey validation was not conducted. The survey had 30 questions, including multiple-choice and short-answer format. Questions were asked about participant demographics, the role that pets play in the physician's relationship with their clients, the role of pets in healthcare access and compliance, and the physician's perception of human-animal interactions. Respondents were asked to expand on their multiple-choice responses to several of the questions to gather more information. Survey participation was voluntary, and all responses were collected anonymously. The survey was deemed exempt by the Tufts University Social, Behavioral, and Educational Research Institutional Review Board (STUDY00005041).

### 2.2 Data analysis

Demographic data and survey responses were analyzed with descriptive statistics using Microsoft Excel. Further statistical analysis investigating subgroup differences among participants was not performed due to the study's limited sample size.

## 3 Results

### 3.1 Demographics

A total of 16 physicians participated in the study and demographic data is presented in Table 1. Most participants were female (*n* = 11; 69%). Most age brackets were represented, and various medical specialties were included. All participants had at least 6 years of experience. Most participants were pet owners themselves, the majority owning dogs, followed by fish/amphibians or livestock.

**Table 1:** Participant demographics (*n* = 16).

Characteristics	*n*	%
Gender		
Female	11	69
Male	5	31
Age		
25–35 years old	1	6
35–45 years old	6	38
45–55 years old	1	6
55–65 years old	5	31
Over 65 years old	3	19
Specialty		
Primary care	1	6
Family medicine	5	31
Internal medicine	8	50
Emergency medicine	1	6
Infectious disease	1	6
Years of experience		
Less than 3 years	0	0
3–5 years	0	0
6–10 years	8	50
11–15 years	0	0
16–20 years	0	0
Over 20 years	8	50
Pet ownership		
Yes	10	63
No	6	38
Species of pet		
Dog	9	56
Fish/amphibian	1	6
Livestock (sheep, goat, cow, chicken)	1	6

### 3.2 Physician-client relationships

A majority (*n* = 9; 56%) of survey participants indicated that most (50%–75%) of the patients they care for live with pets; however, 69% (*n* = 11) indicated that they only occasionally ask their clients about pets in their family, i.e., only when relevant to the patient's presenting condition.

When asked about the impact that asking about pets has on the physicians’ practice and relationship with clients, most participants (*n* = 12; 75%) agreed that it improved their rapport or communication, gave them a better understanding of their patient (*n* = 13; 81%), established common ground with their patient (*n* = 10; 63%), and that their patient appreciated the interest in their pet (*n* = 14; 88%). Only 2 participants (13%) noted that asking about pets had minimal or no impact on their practice and relationship with clients. Despite the indication that pets can serve as a positive bridge for communication between physicians and their clients, only 5 (31%) participants indicated that they routinely ask about their patients’ pets (i.e., with new patients and at periodic wellness visits), and none reported that they very frequently ask about pets (i.e., in all non-emergencies).

Some of the challenges that participants anticipated when asking about their patient's pets were a lack of time (*n* = 8; 50%), the topic not being relevant to the appointment (*n* = 5; 31%), the topic being awkward with no lead in or opportunity (*n* = 1; 6%), and not remembering to ask (*n* = 1; 6%).

### 3.3 Pets as a barrier to treatment access and compliance

When asked whether they believed pets served as a barrier to accessing treatment or services among their clients, only 4 participants (25%) said yes, with the majority responding no (*n* = 11; 69%). However, when participants were asked if any of their patients have declined or resisted recommended treatments/services due to concerns about their pets, the majority of physicians said yes (*n* = 9; 56%), and only 6 participants (38%) indicated they had not experienced resistance to recommended treatment due to pets. The physicians were then asked to estimate the frequency with which they had encountered a patient declining or resisting treatment or services due to concerns about their pets. For this question, a majority of the participants (*n* = 7; 44%) indicated they had encountered it every couple of years, and only 3 participants (19%) indicated they had never encountered this scenario.

Participants were asked to identify all the factors they thought were likely to contribute to their patient's decision to decline or resist recommended treatment/services due to their pets, and the most common factor cited was being unable to find pet care (*n* = 12; 75%). Other factors noted to contribute were monetary concerns (*n* = 3; 19%), emotional stress from being away from their pet (*n* = 7; 44%), and prioritization of the pet's health over the owner's health (*n* = 4; 25%).

When asked if they offer strategies or solutions to clients with pet-related concerns to encourage adherence to treatment guidelines, 11 participants (69%) said yes. However, most physicians (*n* = 10; 63%) replied no when asked if they knew specific strategies/solutions to support clients. Some specific solutions that the participants noted they have suggested to their clients with pet-related concerns were family and friends (*n* = 13; 87%), neighbors (*n* = 11; 73%), animal rescue organizations (*n* = 3; 20%), local veterinary hospitals (*n* = 1; 7%), pet sitters (*n* = 8; 53%), boarding facilities (*n* = 6; 40%), and telehealth appointments (*n* = 1; 7%).

Participants were asked if they believed that a low or no-cost pet boarding or foster program could effectively improve access to care among their patients, and the majority said yes (*n* = 10; 63%). When asked to explain their answer, participants who agreed with the statement cited the high cost of pet care for clients, the benefit for clients who do not have family or friends to help them, and the benefit to hospitalized patients. Participants who disagreed with the statement noted the potential lack of trust in the organization responsible for their pets, the desire to avoid boarding their pet, the ability of most of their clients to make arrangements with family or friends, and lack of transportation.

### 3.4 Human-animal relationships

Participants were asked if they believe that there are psychosocial benefits associated with owning pets, and all participants said yes (*n* = 16; 100%). When asked to explain their answer, reasons included mental health benefits, companionship, reduced loneliness, self-efficacy, personal responsibility, joy or happiness, fulfillment, overall sense of purpose, decreased stress or anxiety, emotional support, and increased physical activity. Most participants indicated that they had discussed the positive effects of companionship and social interaction from pets with their patients (*n* = 13; 87%) as well as the therapeutic effects of pets on anxiety and stress (*n* = 12; 80%).

When asked if they discussed some of the risks associated with pet ownership, only a small portion of participants noted they have discussed concerns about zoonotic diseases (*n* = 3; 20%), an existing or potential animal-related injury (*n* = 2; 13%), or pressure on family or individual resources because of a pet (*n* = 2; 13%). Physicians were asked to rate their comfort level in discussing zoonotic diseases with their clients. Only 1 participant (6%) rated themselves as very comfortable, while 6 (38%) said somewhat comfortable, 4 (25%) said neutral, and 5 (31%) said somewhat uncomfortable.

## 4 Discussion

In this study, we examined the role of pets in healthcare access and compliance as well as patient well-being through the perspective of physicians. Despite pets living in the majority of households in America and playing an essential role in family dynamics and decision-making, this study found that most physicians do not routinely ask about their patient's pets. However, asking about pets was noted by physicians to be a positive factor for communication and rapport. Our findings suggest that physicians routinely asking about pets could be a simple way to build trust and familiarity during appointments in order to foster strong client-patient relationships. This approach may also provide information to facilitate more patient-centered care and personalized lifestyle recommendations to improve patient health outcomes (5). Specifically, asking about pets could be an innocuous way to gather more information about a patient's home life and environment, allowing the patient to open up and reveal clinically relevant information (3, 17). The most common challenges physicians anticipated regarding asking about pets during appointments were lack of time and relevance to the appointment. Adding simple, standardized questions about pets to patient check-in forms or electronic medical records could be an effective solution to ensure the information is available and not forgotten during history taking. This strategy would help identify patients who may be at risk for pet-related barriers to healthcare and subsequently make it easier for providers to support their patients and connect them with resources to improve treatment compliance.

We found that most participants disagreed that pets serve as a barrier to accessing treatment or services among their patients, even though most of them had experienced specific instances of pet-related concerns that interfered with treatment compliance. This finding indicates physicians may underestimate the significance of pets in their patient's lives and the role that pets may play in their patient's healthcare decision-making. Participants recognized that not being able to access pet care was a common reason for a patient to decline or resist treatment. However, the most frequently suggested strategy from participants to address pet-related concerns was relying on the help of family and friends for pet care, followed by relying on neighbors and pet sitters. Pet care options such as pet sitters or boarding facilities are beyond the financial means of many people, particularly when it comes to long-term pet care while an owner is hospitalized. Additionally, relying on family and friends may not be a viable option for pet owners from vulnerable populations who do not have strong support systems or access to family members. Consequently, these findings suggest there is a disparity in physician understanding surrounding the scope and impact of pet ownership and a lack of training regarding how to manage patients with pet-related concerns. The findings also indicate that there is a gap in resources available to patients who need long-term pet care options.

The majority of participants agreed that a low or no-cost boarding facility would be a viable solution for their clients with pet-related concerns. The development of hospital-affiliated pet care assistance programs or community-based pet support networks that provide temporary pet fostering or short-term boarding would serve as a useful resource for providers to offer to their patients. Several animal rescue organizations within the US offer similar temporary pet care programs^1^^–^^5^; however, this solution does not address all concerns reported by the participants in this study, such as the emotional stress pet owners may feel from being away from their pet, the desire to not board a pet long term, and the lack of trust in organizations that may be taking on pet care responsibilities for the owner. Furthermore, patients who need to be away from their pets long term for hospitalization or rehabilitation may experience subsequent adverse mental health outcomes from losing a strong source of emotional support, suggesting that prolonged separation may impact the patient's recovery negatively. For patients, particularly in vulnerable populations, it may be prudent for physicians to inquire about pets when recommending long-term care or hospitalization and assist with pet care arrangements given that lack of access to pet care may impede a patient's ability to adhere to treatment recommendations. Involving case managers and social workers when pet-related concerns arise could also ensure patients receive sufficient support and guidance and are able to be connected to available resources.

All physicians agreed that pets have psychosocial benefits for their patients, and most noted they have discussed these benefits with their clients. However, only a small percentage of the participants reported they had discussed pet-related challenges such as zoonotic disease, injury, or financial responsibilities. These findings suggest that most physicians are aware of the potential benefits of owning pets, which have been discussed in previous literature (3–7), but may underestimate or overlook some of the possible adverse outcomes related to pet ownership that can impact human health outcomes. This study specifically indicates physicians may not fully appreciate the degree to which pet ownership influences personal responsibilities and lifestyle choices which can affect a patient's willingness and ability to seek care for themselves. Integrating One Health oriented modules into continuing medical education for practicing physicians or within medical school curricula that discuss pet-related social determinants of health could serve to better prepare providers to offer patient-centered care by equipping them to communicate more meaningfully with their patients about pet-ownership, including both the positive and negative aspects. An existing model of this kind of education is offered by The University of Washington School of Medicine which provides a clinical elective to medical students that exposes them to multiple clinical applications of One Health including zoonotic diseases, animal-related hazards and injuries, and the power of the human animal bond in medical contexts through gaining hands on experience working within interdisciplinary teams^6^.

### 4.1 Study limitations

The results from this study were analyzed from a small sample size of 16 physicians through convenience sampling. Therefore, these results may not reflect the experiences and opinions of physicians in other geographic areas or patient populations. Given the small sample size of our study, we could not differentiate participant responses based on their subgroup differences, such as specialty area, years of experience, whether the physician was a pet owner or not, or the population served by the physician, such as if they work specifically with vulnerable or low-income populations, which would be an important focus for future research. Participants were asked to report based on their experiences, which could introduce recall or social desirability bias. Additionally, given the recruitment method, there may be a response bias among participants, as individuals with a greater interest in human-animal interactions may be more likely to respond. Furthermore, since we surveyed physicians about their patients, it is possible their responses may not truly reflect what is going on in their patients’ lives, especially if they are not specifically asking about pets. Despite these limitations, this preliminary study highlights the importance of future research focused on larger and more diverse physician populations and that investigate patient experiences by directly surveying patients themselves about their pets.

## 5 Conclusion

Pets play an important role in family dynamics and individual lifestyle choices, including healthcare decision-making. However, healthcare delivery systems are not set up to accommodate the needs of pet owners, especially when it comes to long-term and inpatient care. Additional efforts targeted at understanding and addressing how owning pets may decrease healthcare compliance have the potential to help inform the development of interventions to improve the recognition and management of pet-related concerns by healthcare providers. More research in this area is needed involving professionals from both medical and veterinary backgrounds to address this interdisciplinary issue at the intersection of human patient care and animal well-being. Further studies with larger sample sizes investigating varied physician and patient populations are essential for establishing the need for programs to facilitate better patient care while accommodating pet ownership responsibilities to improve both human and animal health outcomes.

## Data Availability

The raw data supporting the conclusions of this article will be made available by the authors, without undue reservation.

## Footnotes

^1^ https://www.arlboston.org/temporarypethousing/

^5^ https://www.sthuberts.org/temporary-crisis-care#::text=TEMPORARY%20CRISIS%20CARE-,St.,Medical%20emergencies

^6^ https://deohs.washington.edu/cohr/medical-training
