# Supplementary material for: Physician perceptions and understanding of pet ownership in healthcare compliance and patient well-being: a one health investigation
Source: Front Health Serv. 2025 Jul 23;5:1620640. doi: 10.3389/frhs.2025.1620640 (PMC12325250; doi:10.3389/frhs.2025.1620640)
Supplement: Supplementary file 1 [file Table1.docx]

**Supplementary Appendix: Physician Survey**

**Demographic Questions:**

What age category do you fall under?

- 18-25 years old
- 25-35 years old
- 35-45 years old
- 45-55 years old
- 55-65 years old
- Over 65 years old

What is your gender identity?

- Female
- Male
- Non-binary
- Prefer not to answer

How many years of experience do you have working as a physician since graduating medical school (including residency)?

- Less than 3 years
- 3-5 years
- 6-10 years
- 11-15 years
- 16-20 years
- Over 20 years

What is the location of your employment?

What specialty do you fall under?

Do you own any pets currently?

- Yes
- No

If yes to the previous question, what species do you own? Please select all that apply.

- Dog
- Cat
- Rabbit
- Ferret
- Rodent (rat, mouse, hamster, guinea pig)
- Bird
- Reptile
- Fish/Amphibian
- Livestock (sheep, goat, cow, chicken)
- Horse
- Other

If other, please specify:

**Physician-Client Relationship Questions:**

What proportion of the patients/clients you care for live with pets (your best estimate is fine)?

- Very little (0-25%)
- Some (25-50%)
- Most (50-75%)
- Majority (over 75%)
- I don’t know

How often do you ask patients/clients about pets in their family?

- Never
- Occasionally - only when relevant to presenting condition
- Routinely - new patients and at periodic wellness visits
- Very frequently - all non-emergencies

What impact does asking about pets have on your practice and/or relationship with clients? Please select all that apply.

- Improved rapport/communication
- Better understanding of patient
- Establishes common ground
- Patient appreciates interest
- Minimal/none
- Negative/uncomfortable
- Other:

If other, please specify:

What challenges do you have or anticipate having when asking about patients’ pets? Please select all that apply.

- None
- Lack of time
- Not relevant to the appointment
- Awkward (no lead in or opportunity)
- Difficult topic to discuss (uncomfortable)
- Remembering to ask
- Other

If other, please specify:

**Pets as a Barrier to Treatment Access and Compliance Questions:**

Do you believe that pets serve as a barrier to accessing treatment/services among your clients/patients?

- Yes
- No
- I don’t know

Have any of your patients/clients declined or resisted recommended treatment/services (such as inpatient care) due to concerns about their pets?

- Yes
- No
- I don’t know

Please estimate how frequently you encounter patients/clients who decline or resist treatment/services due to concerns about their pets?

- Never
- Once every couple of years
- Once in the past years
- A couple times in the past year
- Once a month
- Over once a month

If you have had patients/clients decline or resist treatment due to concerns about their pets, what factors do you think contributed to their decision? Please select all that apply.

- Monetary concerns
- Unable to find pet care
- Emotional stress from being away from pet
- Prioritization of pet’s health over owner’s health
- Unknown
- N/A
- Other

If other, please specify:

Do you offer strategies/solutions to clients with pet related concerns to encourage adherence to treatment recommendations?

- Yes
- No

Do you know of any strategies/solutions to support clients with pet related concerns to encourage adherence to treatment recommendations?

- Yes
- No

If yes, please explain:

Which of the following solutions have you suggested to your clients with pet related concerns to encourage adherence to treatment recommendations? Please select all that apply.

- Family or friends
- Neighbors
- Animal rescue organizations
- Local veterinary hospital
- Pet sitter
- Boarding facility
- Other

If other, please specify:

Do you believe that a low/no-cost pet-boarding or foster program for patients could serve as an effective solution to improve access to care among your patients?

- Yes
- No
- I don’t know

Please explain your answer:

**Human-Animal Relationships Questions:**

Do you believe there are psychosocial benefits associated with owning pets among patients/clients in your practice?

- Yes
- No
- I don’t know

Please explain your answer:

Which of the following have you discussed with your patients who live with pets? Please select all that apply.

- Positive effect of companionship and social interaction from the pet
- Therapeutic effects of pets on anxiety and stress
- Concern about an infectious zoonotic disease
- Concern about an existing or potential zoonotic injury
- Concern about pressure on family or individual resources because of a pet

How comfortable are you discussing zoonotic diseases with your clients/patients?

- Very comfortable
- Somewhat comfortable
- Neutral
- Somewhat uncomfortable
- Very uncomfortable
